# Variable Surface Glycoprotein RoTat 1.2 PCR as a specific diagnostic tool for the detection of *Trypanosoma evansi *infections

**DOI:** 10.1186/1475-9292-3-3

**Published:** 2004-09-17

**Authors:** Filip Claes, Magda Radwanska, Toyo Urakawa, Phelix AO Majiwa, Bruno Goddeeris, Philip Büscher

**Affiliations:** 1 Faculty of Agriculture and Applied Biological Sciences, K. U. Leuven, Department of Animal Science, Kasteelpark Arenberg 30, 3000 Leuven, Belgium; 2 Prince Leopold Institute of Tropical Medicine, Department of Parasitology, Nationalestraat 155, Antwerpen, Belgium; 3 International Livestock Research Institute (ILRI), Nairobi, Kenya

## Abstract

**Background:**

Based on the recently sequenced gene coding for the *Trypanosoma evansi *(*T. evansi*) RoTat 1.2 Variable Surface Glycoprotein (VSG), a primer pair was designed targeting the DNA region lacking homology to other known VSG genes. A total of 39 different trypanosome stocks were tested using the RoTat 1.2 based Polymerase Chain Reaction (PCR).

**Results:**

This PCR yielded a 205 bp product in all *T. evansi *and in seven out of nine *T. equiperdum *strains tested. This product was not detected in the DNA from *T. b. brucei*, *T. b. gambiense*, *T. b. rhodesiense*, *T. congolense*, *T. vivax *and *T. theileri *parasites. The Rotat 1.2 PCR detects as few as 10 trypanosomes per reaction with purified DNA from blood samples, i.e. 50 trypanosomes/ml.

**Conclusion:**

PCR amplification of the RoTat 1.2 VSG gene is a specific marker for *T. evansi *strains, except *T. evansi *type B, and is especially useful in dyskinetoplastic strains where kDNA based markers may fail to amplify. Furthermore, our data support previous suggestions that some *T. evansi *stocks have been previously misclassified as *T. equiperdum*.

## Background

Surra is an animal disease occurring in Africa, Asia and Latin America, caused by *Trypanosoma evansi*. *T. evansi *belongs to the subgenus *Trypanozoon*, together with *T. equiperdum *and *T. brucei*. The parasite can infect different host species and is mechanically transmitted by different biting flies such as *Tabanidae *and *Stomoxys *as well as by vampire bats such as *Desmodus rotondus *[1]. Camels and horses are very susceptible to the infection and death can occur within weeks or months. Moreover, *T. evansi *infections of cattle and buffaloes usually lead to a pronounced immunosuppression resulting in an increased susceptibility to other opportunistic diseases such as *Pasteurella *and anthrax [2].

Diagnosis of a *T. evansi *infection usually starts with clinical symptoms or the detection of antibodies to *T. evansi*. Conclusive evidence of *T. evansi *infection, however, relies on detection of the parasite in the blood or tissue fluids of infected animals. Unfortunately, parasitological techniques cannot always detect ongoing infections as the level of parasitaemia is often low and fluctuating, particularly during the chronic stage of the disease [3].

As an alternative to parasitological tests, DNA detection based on PCR has been proposed. *Trypanozoon *specific primers have been designed previously: TBR primers which target a 177 bp repeat [4], pMUTEC primers targeting a retrotransposon [5] and ORPHON primers which target the spliced leader sequence [6]. Most of them have been tested on cattle [7,8], water buffaloes [9] or goats [10]. PCR tests for diagnosis of *T. congolense *and *T. vivax *infections exist as well [11]. The development of a PCR test that would be able to differentiate between the different members of the *Trypanozoon *subgenus still remains a challenging issue. For *T. evansi *infections, the only specific test available so far is based on the detection of a kinetoplast DNA sequence [12,13]. However, the existence of dyskinetoplastic trypanosomes such as *T. evansi *RoTat 5.1 [14] and E152 [12] casts doubt about the diagnostic potential of such tests to detect all infections caused by *T. evansi *parasites. Recently, Ventura *et al*. [15] developed a PCR (PCR-Te664) for the detection of *T. evansi *based on a Random Amplified Polymorphic DNA (RAPD) fragment. The taxon specificity of this PCR remains uncertain since it was only tested on nine *T. evansi *strains, one *T. equiperdum*, two *T. b. gambiense *and one *T. b. rhodesiense*. Following evidence that the variable epitope of RoTat 1.2 VSG is expressed by all *T. evansi *strains tested so far [16], and that the gene encoding RoTat 1.2 VSG is present in all *T. evansi *but not in *T. brucei *isolates [17], we designed primers derived from the sequence of this VSG cDNA. In this article we will present and discuss the results obtained with these primers and compare them to the results we obtained using the PCR-Te664.

## Results

### PCR RoTat 1.2 : taxon specificity

**Figure 1:**
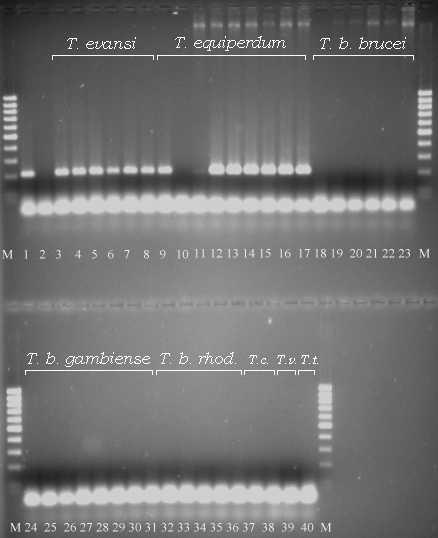
**PCR specificity results for the different *Trypanosoma *(*T.*) species and subspecies in this study. **Lane 1 pos. control RoTat 1.2, Lane 2 neg. control, Lanes 3–8 (*T. evansi*) are, respectively, AnTat 3.1, STIB 816, Zagora I.17, Colombia, Merzouga 56, CAN 86 K; Lanes 9–17 (*T. equiperdum*) are, respectively, AnTat 4.1, BoTat 1.1, OVI, STIB 818, Alfort, Hamburg, SVP, Am. Strain, Can. Strain ; Lanes 18–23 (*T.b.brucei*) are, AnTat 1.8, AnTat 2.2, AnTat 5.5, KETRI 2494, TSW 196, STIB 348; Lanes 24–31 (*T.b.gambiense*) are, respectively, AnTat 9.1, AnTat 11.6, AnTat 22.1, NABE, SEKA, ABBA, LIGO, LiTat 1.6; Lanes 32–36 (*T.b. rhodesiense*) are STIB 884, STIB 850, AnTat 25.1/S, Etat 1.2/S, AnTat 12.1/S ; Lanes 37–38 (*T. congolense*) are IL1180, TRT 17; Lane 39 (*T. vivax*) is ILRAD 700 and Lane 40 (*T. theileri*) is MELSELE ; Lanes M 100 bp molecular marker (MBI Fermentas, Germany).

The 39 different trypanosome stocks used in this study are listed in Table 1 [see Additional file 1]. They were derived from a wide range of hosts and from distinct geographical locations. In all PCR runs, RoTat 1.2 DNA was used as a positive control. As shown in Figure 1, the RoTat 1.2 PCR yielded a 205 bp amplicon in the positive control (lane 1) as well as in all other *T. evansi *populations (lanes 3–8). Moreover, the same fragment was found in seven out of the nine *T. equiperdum *populations tested. The *T. equiperdum *BoTat 1.1 was PCR negative (lane 10), while the *T. equiperdum *OVI strain yielded a PCR product shorter than 205 bp (lane 11) probably due to mispriming. All other tested trypanosome populations, including six *T. b. brucei*, eight *T. b. gambiense*, five *T. b. rhodesiense*, two *T. congolense*, one *T. vivax *and one *T. theileri*, were negative. (lanes 18–40). As a negative control, a PCR-mix without template DNA was included (lane 2). Sequencing of the positive samples revealed that all amplicon were identical (data not shown). The weak band in OVI did not yield sufficient material to enable sequencing.

### PCR RoTat 1.2 : analytical sensitivity

**Figure 2:**
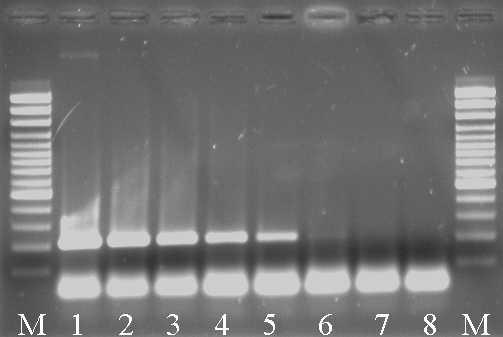
**Analytical sensitivity of the RoTat 1.2 PCR. **Lanes M 100 bp molecular marker (MBI Fermentas, Germany); lane 1: 10^5 ^trypanosomes, lane 2: 10^4 ^trypanosomes, lane 3: 10^3 ^trpyanosomes, lane 4: 10^2 ^trypanosomes, lane 5: 10 trypanosomes, lane 6: 1 trypanosome, lane 7: 0.1 trypanosome, lane 8: negative control.

A tenfold dilution series (10^5 ^trypanosomes down to 1 trypanosome per 200 μl sample) of RoTat 1.2 trypanosomes in mouse blood was prepared to determine the analytical sensitivity of the PCR. As shown in figure 2, the PCR was able to detect as few as 10 trypanosomes per PCR reaction, which corresponds with a lower detection limit of 50 trypanosomes per ml. In principle, this limit can still be lowered if a blood sample of 200 μl extracted with the QIAamp DNA mini kit is eluted in less than 200 μl.

### PCR-Te664 : taxon specificity

To evaluate the RoTat 1.2 diagnostic system alongside other published methods, we compared our method to the PCR-Te664 method as published by Ventura et al. [15] using the same trypanosome stocks. The PCR-Te664 method yielded the expected amplicon in all seven *T. evansi *strains and in seven out of nine *T. equiperdum*. As with the RoTat 1.2 PCR only *T. equiperdum *strains OVI and BoTat 1.1 remained negative. Unexpectedly, four out of six *T. b. brucei *(AnTat 2.2, AnTat 5.2, TSW 196 and KETRI 2494) and two *T. b. gambiense *type II strains (ABBA and LIGO) tested positive in this PCR (data not shown).

## Discussion

This study was initiated to develop a specific PCR test that would be able to distinguish *T. evansi *from the other members of the *Trypanozoon *subgenus. The study is an extension of the initial observation that the RoTat 1.2 VSG gene only is found in *T. evansi *and not in *T. brucei *strains [17]. This study mainly focused on the presence and expression of the RoTat 1.2 VSG gene in *T. evansi *rather than the use of this VSG in diagnosis of Salivarian trypanosomes.

Previously, other research groups have used VSG genes as target sequences for PCR detection of *T.b. gambiense *infections (sleeping sickness). In these studies, five different primers derived from VSG genes, AnTat 11.17, LiTat 1.3, 117, 2 K and U2 were used in PCR screening of different trypanosome populations, originating from distinct geographical locations [18-20]. AnTat 11.17 based PCR tests were capable of distinguishing *T.b. gambiense *from *T.b. brucei *parasites from most foci of sleeping sickness in countries such as Nigeria, Cameroon, Côte d'Ivoire, R. P. Congo/Brazza. and Sudan. However, populations originating from the Moyo focus in North-west Uganda and from Cameroon were shown to be negative in AnTat 11.17 and in LiTat 1.3 (2 K) PCRs respectively. According to Bromidge *et al*. [18], this might be due to antigenic variation and genetic evolution of the VSG genes. On the other hand, the presence of 117 and U2 genes was shown to be a common feature among all *T. brucei *populations tested. In *T. evansi*, a similar phenomenon may occur in certain Kenyan isolates. A recent study by Ngaira *et al*. [21] pointed out that some *T. evansi *stocks in the Isiolo district in Kenya seem to lack the Rotat 1.2 VSG gene. It is believed that these stabilates belong to the *T. evansi *type B group. So far, this type of *T. evansi *has only been observed in this specific region in Kenya [22,23]. To our knowledge, all other *T. evansi *isolated elsewhere, are from the classical *T. evansi *type A group. Thus, we assume that, except for these few Kenyan strains belonging to the type B group, our PCR is specific for *T. evansi*.

Compared to the PCR-Te664 presented by Ventura *et al*. [15], the PCR RoTat 1.2 seems to have a higher taxon specificity, since no reaction with *T. b. brucei*, nor with *T. b. gambiense *type II was observed. However, regarding *T. equiperdum*, both PCR test positive for the same seven *T. equiperdum *strains and are both negative for the BoTat 1.1 and OVI strains. Since the RAPD fragment (AF397194) shares no homology with the Rotat 1.2 VSG gene (AF317914) and is not found within the expression site of trypanosomes, both sequences can be considered as independent molecular markers. Based on the observations with both markers, it appears that on the genomic level the Botat 1.1 and the OVI strains are different from the other *T. equiperdum *and *T. evansi *strains. The observed analytical sensitivity with the RoTat 1.2 PCR is comparable to what was reported for the Te664 PCR (25 cells per reaction) [15].

The presence of a RoTat 1.2 specific DNA sequence in some *T. equiperdum *strains corresponds with the serological evidence that rabbits experimentally infected with these strains develop RoTat 1.2 specific lytic antibodies within 30 days post infection [24]. In contrast, rabbits infected with the BoTat 1.1 clone and the OVI strain, which are negative in the present PCR, did not produce specific antibodies to the RoTat 1.2 clone when tested in immune trypanolysis. This might be explained by the loss of the RoTat 1.2 gene in the OVI and the BoTat 1.1 strain. It is also possible that there has been a sequence drift at the sites where these primers could bind. However, we hypothesize that RoTat 1.2 VSG truly is *T. evansi *specific and that RoTat 1.2 PCR positive *T. equiperdum *strains are actually *T. evansi *and not *T. equiperdum*. Indeed, in a previous molecular characterization study using Random Amplified Polymorphic DNA (RAPD) and the Multiplex-endonuclease Genotyping Approach (MEGA) it appeared that the *T. equiperdum *collection is not as homogenous as previously believed and that the generally followed concept that *T. equiperdum *is very closely related to *T. evansi *and more distant from *T. b. brucei*, seems incorrect. From the cluster analysis on the available strains, it appeared that only two clusters can be identified: a homogeneous *T. evansi*/*T. equiperdum *cluster and a more heterogeneous *T. b. brucei*/*T. equiperdum *cluster [25]. Interestingly, all strains of that homogeneous *T. evansi*/*T. equiperdum *cluster are all PCR RoTat 1.2 VSG positive while the strains found in the more heterogeneous *T. b. brucei*/*T. equiperdum *cluster, in casu BoTat 1.1 and OVI are PCR RoTat 1.2 VSG negative.

## Conclusions

PCR amplification of the RoTat 1.2 VSG gene is a specific marker for *T. evansi *strains, except *T. evansi *type B, and is especially useful in dyskinetoplastic strains where kDNA based markers may fail to amplify. Furthermore, our data support previous suggestions that some *T. evansi *stocks have been previously misclassified as *T. equiperdum*.

## Methods

### Trypanosome populations

A total of 39 different trypanosome populations were used in this study. They belong to 39 stocks and six species, isolated from a variety of host species at distinct geographical locations (Table 1 [see Additional file 1]). Only three *T. equiperdum *strains, BoTat 1.1, OVI and STIB 818 are well documented, i.e. known origin and host. The other six are putative *T. equiperdum*, based on publications or on their use as reference strains in different national dourine reference laboratories [26-30].

### Preparation of trypanosome DNA

Procyclic trypanosome populations were grown *in vitro *in Cunningham's medium [31] and in the Kit for In Vitro Isolation (KIVI) [32]. Pure procyclic trypanosomes were obtained by repeated centrifugation (20 min., 2000 g) and sediment washes with Phosphate Glucose Sacharose buffer (PGS) (38 mM Na_2_HPO_4_.2H_2_0, 2 mM NaHPO_4_, 80 mM glucose, 100 mM sacharose, pH 8.0). Bloodstream form trypanosomes were expanded in mice and rats and were purified from the blood by di-ethyl-amino-ethyl (DEAE) chromatography [33], followed by repeated centrifugation (20 min., 2000 g) and sediment washes with Phosphate Buffered Saline Glucose (PSG) (38 mM Na_2_HPO_4_.2H_2_0, 2 mM NaHPO_4_, 80 mM glucose, 29 mM NaCl, pH 8.0). Trypanosome sediments were subsequently stored at -80°C.

Twenty μl of trypanosome sediment (approximately 2.10^7 ^cells) were resuspended in 200 μl of Phosphate Buffered Saline (PBS) (8.1 mM Na_2_HPO_4_.2H_2_0, 1.4 mM NaHPO_4, _140 mM NaCl, pH 7.4) and the trypanosome DNA was extracted using the commercially available QIAamp DNA mini kit (Westburg, Leusden, The Netherlands), resulting in pure DNA in 200 μl of TE buffer. The typical yield of DNA extracted from a 20 μl pellet was 150 ng/μl or 30 μg total DNA. The extracts obtained were diluted 200 times in water and divided into aliquots of 2 ml in microcentrifuge tubes for storage at -20°C.

For trypanosome dilution series, 180 μl of each heparinized blood sample were mixed with an equal volume of the Qiagen AS-1 storage buffer and subsequently extracted using the QIAamp DNA blood mini kit (Westburg, Leusden, The Netherlands) resulting in 200 μl of extracted DNA in Millipore water. Manipulation was performed according to the manufacturer's instructions.

### PCR RoTat 1.2

Primers were derived from the RoTat 1.2 VSG sequence (AF317914), recently cloned and sequenced by Urakawa *et al*. [17]. Using DNA sequence homology search programs to interrogate databases at TIGR (The Institute for Genomic Research) and GenBank, primer sequences were identified within the region (608–812 bp) lacking homology with any other known VSG sequence present in the databases. Primer sequences (in 5'-3' direction) and annealing temperatures were as follows: RoTat 1.2 Forward GCG GGG TGT TTA AAG CAA TA, T_ann. _59°C and RoTat 1.2 Reverse ATT AGT GCT GCG TGT GTT CG, T_ann. _59°C.

Twenty μl of extracted DNA were mixed with 30 μl of a PCR-mix containing: 1 U Taq DNA recombinant polymerase (Promega, UK), PCR buffer (Promega, UK), 2.5 mM MgCl_2 _(Promega, UK), 200 μM of each of the four dNTPs (Roche, Mannheim, Germany) and 0.8 μM of each primer (Gibco BRL, UK).

All amplifications were carried out in a Biometra^® ^Trio-block thermocycler. Cycling conditions were as follows: denaturation for 4 min. at 94°C, followed by 40 amplification cycles of 1 min. denaturation at 94°C, 1 min. primer-template annealing at 59°C and 1 min. polymerization at 72°C. A final elongation step was carried out for 5 min. at 72°C.

Twenty μl of the PCR product and ten μl of a 100 bp size marker (MBI Fermentas, Germany) were subjected to electrophoresis in a 2 % agarose gel (25 min. at 100 V). Gels were stained with ethidium bromide (0.5 μg/ml) (Sigma, USA) and analyzed on an Imagemaster Video Detection System (Pharmacia, UK).

### PCR Te-664

PCR on purified DNA samples was performed using primers and PCR conditions according to Ventura *et al*. [15]. Only the *Taq *DNA polymerase was purchased from another distributor, i.e. Promega (UK) instead of Gibco BRL (UK).

## Competing interests

None declared.

## Authors' contributions

FC carried out the molecular work and drafted the manuscript. MR and TU participated in the molecular analysis. PM, BG and PB participated in the design and co-ordination of the study. All authors read and approved the final manuscript.

## Supplementary Material

Additional File 1 Table 1. Data on the different *Trypanosoma *(*T*.) populations used in this study Click here for file
