# Supplementary material for: Variable Surface Glycoprotein RoTat 1.2 PCR as a specific diagnostic tool for the detection of Trypanosoma evansi infections
Source: Kinetoplastid Biol Dis. 2004 Sep 17;3:3. doi: 10.1186/1475-9292-3-3 (PMC521498; doi:10.1186/1475-9292-3-3)
Supplement: Additional File 1 — Table 1. Data on the different Trypanosoma (T.) populations used in this study [file 1475-9292-3-3-S1.doc]

| **N°** | **Species** | **Clone/strain** | **ITMAS** | **Origin** | **Form** | **Host/Vector** |
| --- | --- | --- | --- | --- | --- | --- |
| 1 | *T. evansi* | RoTat 1.2 | 020298 | Indonesia | bloodstream | buffalo |
| 2 | *T. evansi* | AnTat 3.1 | 070799 | South America | bloodstream | capybara |
| 3 | *T. evansi* | STIB 816 | 140799C | P. R. China | bloodstream | camel |
| 4 | *T. evansi* | Zagora I.17 | 040399B | Morocco | bloodstream | camel |
| 5 | *T. evansi* | Colombia | 150799 | Colombia | bloodstream | horse |
| 6 | *T. evansi* | Merzouga 56 | 120399D | Morocco | bloodstream | camel |
| 7 | *T. evansi* | Can 86 K | 140799B | Brazil | bloodstream | dog |
| 8 | *T. equiperdum* | AnTat 4.1 | 210983A | Unknown | bloodstream | unknown |
| 9 | *T. equiperdum* | BoTat 1.1 | 240982A | Morocco | bloodstream | horse |
| 10 | *T. equiperdum* | OVI | 241199C | South Africa | bloodstream | horse |
| 11 | *T. equiperdum* | STIB 818 | 010999 | P. R. China | bloodstream | Horse |
| 12 | *T. equiperdum* | Alfort | 241199A | Unknown | bloodstream | Unknown |
| 13 | *T. equiperdum* | Hamburg | 251199A | Unknown | bloodstream | Unknown |
| 14 | *T. equiperdum* | SVP | 241199B | Unknown | bloodstream | Unknown |
| 15 | *T. equiperdum* | Am. Strain | 220101 | Unknown | bloodstream | Unknown |
| 16 | *T. equiperdum* | Can. Strain | 290101 | Unknown | bloodstream | Unknown |
| 17 | *T. b. brucei* | AnTat 1.8 | 121296A | Uganda | bloodstream | Bushbuck |
| 18 | *T. b. brucei* | AnTat 2.2 | 100297B | Nigeria | procyclic | Tsetse fly |
| 19 | *T. b. brucei* | AnTat 5.2 | 220197 | Gambia | bloodstream | Cattle |
| 20 | *T. b. brucei* | KETRI 2494 | 270881 | Kenya | procyclic | Tsetse fly |
| 21 | *T. b. brucei* | TSW 196 | 300500A | Côte d'Ivoire | bloodstream | Unknown |
| 22 | *T. b. brucei* | STIB 348 | 250500B | Tanzania | bloodstream | Unknown |
| 23 | *T. b. gambiense* | AnTat 9.1 | 010399A | Cameroon | bloodstream | Human |
| 24 | *T. b. gambiense* | AnTat 11.6 | 280584 | R. D. Congo | bloodstream | Human |
| 25 | *T. b. gambiense* | AnTat 22.1 | 110280 | Congo/Brazza. | bloodstream | Human |
| 26 | *T. b. gambiense* | NABE | 201295 | R. D. Congo | procyclic | Human |
| 27 | *T. b. gambiense* | SEKA | 221295 | R. D. Congo | procyclic | Human |
| 28 | *T. b. gambiense II* | ABBA | 190600A | Côte d'Ivoire | bloodstream | Human |
| 29 | *T. b. gambiense II* | LIGO | 190600B | Côte d'Ivoire | bloodstream | Human |
| 30 | *T. b. gambiense* | LiTat 1.6 | 121296 | Côte d' Ivoire | bloodstream | Human |
| 31 | *T. b. rhodesiense* | STIB 884 | 150399A | Uganda | bloodstream | Human |
| 32 | *T. b. rhodesiense* | STIB 850 | 050399C | Uganda | bloodstream | Human |
| 33 | *T. b. rhodesiense* | AnTat 25.1/S | 300381A | Rwanda | bloodstream | Human |
| 34 | *T. b. rhodesiense* | Etat 1.2/S | 140476A | Uganda | bloodstream | tsetse fly |
| 35 | *T. b. rhodesiense* | AnTat 12.1/S | 120680B | Rwanda | bloodstream | human |
| 36 | *T. congolense* | IL 1180 | 270197 | Tanzania | bloodstream | lion |
| 37 | *T. congolense* | TRT 17 | 020699 | Zambia | bloodstream | cattle |
| 38 | *T. vivax* | ILRAD 700 | 190100 | Nigeria | bloodstream | cattle |
| 39 | *T. theileri* | MELSELE | 020299 | Belgium | procyclic | cattle |
